# Real-world six-month outcomes in patients switched to faricimab following partial response to anti-VEGF therapy for neovascular age-related macular degeneration and diabetic macular oedema

**DOI:** 10.1038/s41433-024-03364-y

**Published:** 2024-10-11

**Authors:** Grace A. Borchert, Christine A. Kiire, Niamh M. Stone, Handan Akil, Theodora Gkika, M. Dominik Fischer, Kanmin Xue, Jasmina Cehajic-Kapetanovic, Robert E. MacLaren, Peter Charbel Issa, Susan M. Downes, Samantha R. De Silva

**Affiliations:** 1 Nuffield Laboratory of Ophthalmology, Nuffield Department of Clinical Neurosciences, University of Oxford, Oxford, UK; 2 Oxford Eye Hospital, Oxford University NHS Foundation Trust, Oxford, UK; 3 Department of Ophthalmology, Technical University Munich, Munich, Germany

**Keywords:** Health care, Therapeutics

## Abstract

**Background:**

Landmark studies reported on faricimab efficacy and safety predominantly in treatment naïve patients, but outcomes following switch from other anti-VEGF therapies are lacking. We evaluated patients switched to faricimab who had previously shown a partial response to other anti-VEGF injections for neovascular age-related macular degeneration (nAMD) and diabetic macular oedema (DMO).

**Methods:**

Retrospective study at the Oxford Eye Hospital. Patients switched to faricimab from January to April 2023 with six months follow-up were identified via electronic medical records.

**Results:**

A total of 116 patients (151 eyes) were included. In 88 patients with nAMD (107 eyes), mean visual acuity remained stable: 62±17 ETDRS letters at baseline; 62±18 at six months (*p* > 0.05). Central subfield thickness (CST) reduced from 294 ± 73 μm to 270 ± 53 μm (*p* < 0.05) at six months. Subretinal or intraretinal fluid was present in 102 eyes (95%) at baseline and 75 eyes (70%) at follow-up (*p* < 0.05). Pigment epithelial detachment height decreased from 233 ± 134 μm to 188 ± 147 μm (*p* < 0.05). Mean treatment interval increased by 1.7 weeks (*p* < 0.05) and was extended in 61 eyes (57%) at six months. In 28 patients with DMO (44 eyes), visual acuity remained stable: 69 ± 15 letters at baseline; 70±15 at six months (*p* > 0.05). CST reduced from 355 ± 87 μm to 317 ± 82 μm (*p* < 0.05). Mean treatment interval increased by 1.4 weeks (*p* < 0.05) and was extended in 21 eyes (46%) by six months.

**Conclusions:**

Switching to faricimab in treatment resistant eyes led to improved anatomical response and extended treatment interval in a significant proportion of patients. Ongoing review of real-world data will inform longer-term outcomes of safety and effectiveness.

## Introduction

Neovascular age-related macular degeneration (nAMD) and diabetic macular oedema (DMO) are leading causes of visual impairment in the developed world [1, 2]. In both conditions, angiogenic and inflammatory growth factors, including vascular endothelial growth factor (VEGF) and angiopoietin-2 (Ang-2), are upregulated. VEGF regulates angiogenesis, amongst other functions, while pathological upregulation of Ang-2 induces vascular leakage and abnormal blood vessel structure [3]. Anti-VEGF agents have become the mainstay of treatment for nAMD, DMO, and macular oedema caused by retinal vein occlusions. In most cases, repeated intravitreal injections of anti-VEGF agents are required, leading to a significant treatment burden for patients and cost for healthcare systems [4].

Faricimab (Vabysmo^TM^; Roche/Genentech, Switzerland) is a dual inhibitor of VEGF and Ang-2, approved by the Food and Drug Administration (FDA, February 2022, USA), Medicines and Healthcare products Regulatory Agency (MHRA, May 2022, UK), and the European Medicine Agency (EMA, October 2022, EU) [5–7]. It is the first bi-specific, humanised, IgG monoclonal antibody that is given by intravitreal injection for intraocular use [8]. Phase 2 and 3 AVENUE, STAIRWAY, TENAYA and LUCERNE clinical trials in treatment-naïve nAMD populations showed best corrected visual acuity (BCVA) gains and anatomical improvements with non-inferiority to aflibercept [9–11]. In the predominantly treatment naïve DMO participants of the BOULEVARD, YOSEMITE and RHINE trials, similar beneficial outcomes were demonstrated [12, 13].

These trials also showed that the treatment interval could be extended in a significant proportion of patients in both cohorts, with approximately 45% of patients achieving 16-weekly dosing in the TENAYA and LUCERNE trials at 48 weeks, and 51–53% in the YOSEMITE and RHINE trials. In addition to cost and treatment burden, repetitive anti-VEGF injections increase the risk of complications such as endophthalmitis, immunoreactivity and tachyphylaxis [14, 15]. Reduced injection frequency is, therefore, desirable.

Comprehensive real-world data on faricimab outcomes are currently lacking, although published studies to date do report favourable outcomes. Real world studies are needed to understand the effectiveness of faricimab in patients switched from other anti-VEGF agents since landmark studies characterised faricimab efficacy in predominantly treatment naïve populations [16]. Response to anti-VEGF therapy is known to vary, with treatment resistance, persistent disease activity, or non-response reported in up to 30% of patients [17]. This may in part be explained by under-dosing in the real world [18], possibly due to challenges regarding clinic capacity and patients’ ability to attend frequent appointments. However, true incomplete or partial anti-VEGF response is well documented in a proportion of patients and some genetic associations affecting treatment response have been reported [14, 15, 19].

We aimed to investigate the effectiveness and safety of faricimab in patients with nAMD and DMO, who previously had a partial response to other anti-VEGF agents. We report 6-month outcomes in this cohort.

## Methods

A retrospective, observational study was undertaken at the Oxford Eye Hospital, United Kingdom. Medical records were screened for patients switched to faricimab therapy for nAMD and DMO between January and April 2023. This study was approved by the Oxford University Hospitals clinical governance and audit department. Eyes included were those switched to faricimab from treatment with other anti-VEGF agents due to partial response. This was defined as a reduction in disease activity compared to OCT at initiation of anti-VEGF therapy, but with persistence of intraretinal fluid or subretinal fluid (in nAMD and DMO), or subretinal hyperreflective material or pigment epithelial detachment (in nAMD), despite four to six weekly treatment with anti-VEGF injections.

Eyes were included if visual acuity (VA) with usual spectacles if worn, and OCT data were available at baseline and at six months (±6 weeks) (Supplementary Fig. 1). On switching, all patients with nAMD were prescribed faricimab at the same treatment interval as their previous anti-VEGF agent, i.e. four or six weekly injection without a loading course. For DMO, the decision to prescribe a loading course of faricimab injections (one injection every four weeks for four injections), or injections at a matched treatment interval to the previous anti-VEGF agent was at the discretion of the responsible clinician. Subsequently, eyes were treated according to a treat and extend protocol (Supplementary Fig. 2).

Eyes were excluded from this analysis if they had an ophthalmic procedure affecting vision (such as cataract surgery or YAG capsulotomy) during the six month follow-up period, were lost to follow up, or had a gap in the intended treatment course of more than 8 weeks due to illness or cancelled appointments (Supplementary Fig. 1). Patients switched away from faricimab treatment were considered as a separate subset in the study.

Data extracted included the anti-VEGF agent prior to faricimab switch, previous treatment interval and number of intravitreal injections given. Visual acuity with usual spectacles, central subfield thickness (CST) and presence/absence of subretinal fluid (SRF) and intraretinal fluid (IRF) on optical coherence tomography (OCT) scans were recorded at baseline and at six months. In nAMD patients, pigment epithelial detachment (PED) maximal height was measured, if present, at baseline and at six months. The planned treatment interval at six months and the number of faricimab injections given within the six month observational period were also recorded, as well as the occurrence of any adverse events. Disease activity in nAMD was evaluated by assessing the proportion of patients with >50 μm decrease in CST (improvement), <50 μm change (stable) and >50 μm increase (deterioration) [20]. In DMO, this was evaluated by assessing patients with >10% decrease in CST (improvement), <10% change (stable) and >10% increase (deterioration) [21].

Data were analysed with GraphPad Prism 9 (GraphPad software, San Diego, CA). A *t* test was used for comparisons between baseline and six months. Mean (±standard deviation) values are presented and a *p* value of <0.05 was considered to be statistically significant.

## Results

Data from 116 patients (151 eyes) were included in the analyses. Baseline patient characteristics are summarised in Table 1.

**Table 1:** Characteristics of patients at baseline.

	nAMD	DMO
Number of patients	88	28
Number of eyes	107	44
Age at baseline (mean, SD) in years	79 (7)	64 (12)
Females (%)	52 (59%)	9 (32%)
Males (%)	36 (41%)	19 (68%)
Switched from aflibercept (%)	102 (95%)	44 (100%)
Number of injections prior to switch (mean, SD)	26 (18)	22 (11)
Baseline treatment interval (mean, SD) in weeks	5.2 (1.7)	5.2 (1.8)
Baseline BCVA (SD) ETDRS letters	62 (17)	69 (15)
Central subfield thickness (mean, SD) in microns	294 (73)	355 (87)
Subretinal fluid at baseline (n, %)	58 (54%)	0 (0%)
Intraretinal fluid at baseline (n, %)	81 (76%)	44 (100%)
Either SRF or IRF at baseline (n,%)	102 (95%)	44 (100%)

### nAMD

Eighty-eight patients (107 eyes) were included. The mean (±SD) age at baseline was 79 (±7) years. Fifty-nine percent were female. Prior to faricimab switch, a mean of 26 (±18) anti-VEGF injections had been given per eye, with most recent mean treatment interval of 5.2 (±1.7) weeks. The majority of eyes (102 eyes; 95%) were switched from aflibercept and 5 eyes (5%) were switched from brolucizumab. At the time of switch, subretinal or intraretinal fluid was present in 102 eyes (95%), and a persistent pigment epithelial detachment in 2 eyes (2%).

### DMO

Twenty-eight patients (44 eyes) were included. The mean age was 64 (±12) years and 31% were female. Prior to faricimab switch, a mean of 22 (±11) injections had been given per eye, with most recent treatment interval of 5.2 (±1.8) weeks. All patients were switched from aflibercept to faricimab, and persistent disease activity (intraretinal fluid) was present in all eyes (100%) at baseline.

### nAMD

**Fig. 1:**
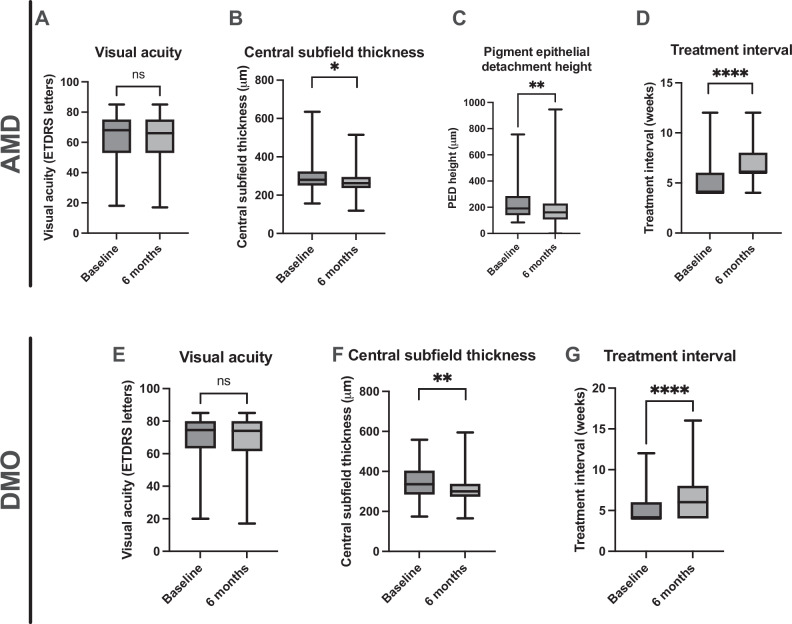
Outcomes at six months in eyes with neovascular AMD and diabetic macular oedema switched to faricimab. Visual acuity (**A**), central subfield thickness (**B**), pigment epithelial detachment height (**C**) and treatment interval (**D**) are shown at baseline and six months in eyes with neovascular AMD. Visual acuity (**E**), central subfield thickness (**F**), and treatment interval (**G**) at baseline and six months are shown in eyes with DMO. ns not significant, *p < 0.05, **<0.01, ****<0.0001.

**Fig. 2:**
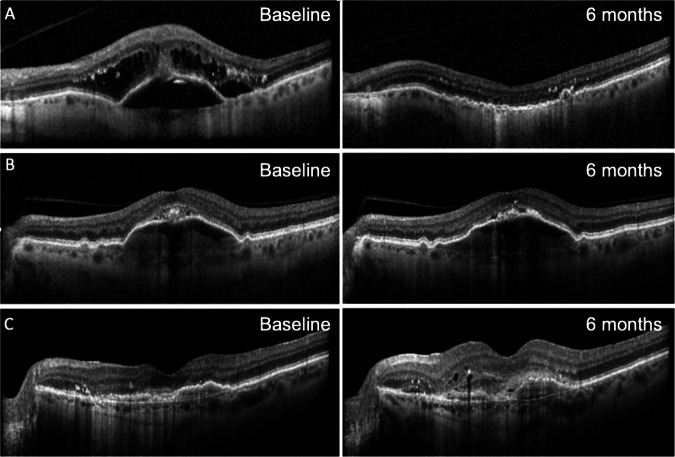
OCT scans illustrating the range of responses seen following switch to faricimab in eyes with nAMD. **A** Improved response: a 74-year-old male previously treated with a total of 35 aflibercept injections with most recent treatment interval of 4 weeks, had a VA of 50 ETDRS letters and CST of 635 μm at baseline. Following switch to faricimab, this improved to 75 ETDRS letters and CST of 208 μm at six months, with resolution of PED, subretinal and intraretinal fluid and treatment interval was extended to 6 weekly. **B** Stable response: A 73-year-old male previously treated with a total of 4 aflibercept injections last given 4 weeks previously, had a VA of 68 ETDRS letters and 285 μm at baseline. Following switch to faricimab, at six months VA improved to 79 ETDRS letters, CST remained at 285 μm, and treatment interval remained at 4 weekly. **C** Disease progression: a 78-year-old female previously treated with a total of 20 aflibercept injections, with last treatment interval of 4 weeks. She had a VA of 69 ETDRS letters and CST of 326 μm at baseline with mild intraretinal fluid (IRF) and subretinal hyperreflective material (SHRM). At six months, VA decreased to 50 ETDRS letters and CST had increased to 392 μm with more IRF and SHRM evident on OCT scan, despite a 4 weekly faricimab treatment interval.

Best-corrected visual acuity remained stable over the follow-up period at 62 (±17) ETDRS letters at baseline and 62 (±18) letters at six months (*p* > 0.5), Fig. 1A. CST decreased from a mean of 294 μm (±73) at baseline to 270 μm (±53) at six months (*p* < 0.05), Fig. 1B. Seventeen eyes (16%) showed improvements in CST ( > 50 μm CST decrease), 88 eyes (82%) were stable (<50 μm change) and 2 eyes (2%) worsened (>50 μm increase). Ninety eyes had a PED at baseline. In nine of these eyes (10%), the PED completely resolved at six months. There was a significant decrease in mean PED height from 233 μm (±134) at baseline to 188 μm (±147) at six months (*p* < 0.05), Fig. 1C. The number of eyes with SRF reduced from 58 (54%) at baseline to 42 (39%) at six months, and from 81 (76%) to 59 (55%) with IRF. Twenty-seven eyes (25%) showed resolution of either SRF or IRF. The range of responses seen following faricimab switch is illustrated in Fig. 2.

Treatment interval was extended from a mean of 5.2 (±1.7) weeks at baseline to 6.9 (±2.3) weeks at six months follow up (1.7 weeks difference, *p* < 0.05), Fig. 1D. The treatment interval was extended in 61 eyes (57%), remained unchanged in 29 eyes (27%), and was decreased in 17 eyes (16%). The mean number of injections given in the six month follow-up period was 5 (±1) injections per eye. Twenty-seven eyes (25%) received one injection of faricimab every four weeks for four injections following the switch.

### DMO

**Fig. 3:**
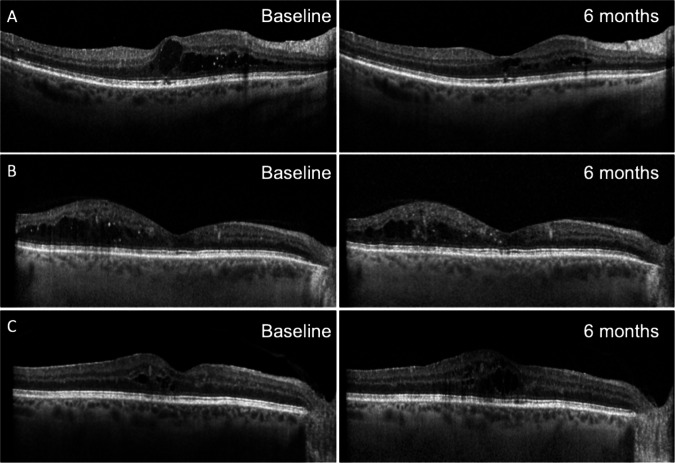
OCT scans illustrating the range of responses seen following switch to faricimab in eyes with DMO. **A** Improved response: A 72-year-old male previously treated with a total of 32 aflibercept injections last given 4 weeks previously, had a VA of 82 ETDRS letters and CST of 403 μm at baseline. Following switch to faricimab, at six months this improved to 289 μm, visual acuity remained stable at 82 ETDRS letters and treatment continued with 4 weekly injections. **B** Stable response: a 62-year-old male previously treated with a total of 17 aflibercept injections last given eight weeks previously, had a VA of 64 ETDRS letters and CST of 232 μm at baseline. Following switch to faricimab, at six months CST was similar at 233 μm, VA was 60 ETDRS letters and treatment interval was eight weekly. **C** Disease progression: a 74 year-old male previously treated with a total of 41 aflibercept injections last given five weeks previously, had a VA of 81 ETDRS letters and CST of 277 μm at baseline. Following switch to faricimab, at six months CST was increased at 339 μm, VA was stable at 83 ETDRS letters and treatment interval with faricimab was four weekly.

A similar trend in VA, CST and treatment interval was seen in patients with DMO. VA remained stable at 69 (±15) ETDRS letters at baseline compared to 70 (±15) ETDRS letters at six months (*p* > 0.05), Fig. 1E. CST decreased from 355 μm (±87) at baseline to 317 μm (±82) at six months (*p* < 0.05), Fig. 1F. Eighteen eyes (38%) showed improvement in CST (>10% decrease in CST), 24 eyes (51%) remained stable (within 10% change in CST) and 2 eyes (4%) worsened (>10% increase in CST) compared to baseline [22]. Intraretinal fluid resolved in four eyes (9%) at six months. The range of responses seen following faricimab switch is illustrated in Fig. 3.

We investigated the effect of glycaemic control (HbA1c) on treatment response. An HbA1c result within six months of switch to faricimab was available in 25/28 patients (89%), and mean HbA1c was 8.3%. There was no correlation between HbA1c and percentage change in CST (R^2^ squared = 0.004; Supplementary Fig. 3).

Mean treatment interval was extended from 5.2 (±1.8) weeks at baseline to 6.6 (±2.5) weeks at six months (1.4 week difference, *p* < 0.05), Fig. 1G. Treatment interval was extended in 21 eyes (48%), remained unchanged in 18 eyes (41%), and was shortened in 5 eyes (11%). The mean number of injections given in the six month follow-up period was 5.7 (±1) per eye. Thirty-four eyes (78%) were given one injection of faricimab every four weeks for four injections following the switch.

## Adverse events and switch from faricimab

There were no reported complications such as endophthalmitis, retinal vasculitis or severe intraocular inflammation events during the follow-up period. As detailed in supplementary Figure 1, one patient had a stroke three months after switching from aflibercept to faricimab. This patient had predisposing risk factors for a stroke, including hypertension, atrial fibrillation and hypercholesterolaemia. A retinal detachment developed in one eye (two months after switching and three weeks after last intravitreal injection), most likely secondary to posterior vitreous detachment, and these eyes were excluded from analysis.

Two patients (three eyes) were switched from faricimab during the six month follow-up. One patient (one eye) with nAMD requested to be switched back to aflibercept as they reported worsening vision, with a decrease of six ETDRS letters recorded after three faricimab injections, OCT scan showed reduced SRF but persistent IRF in this case. Another patient with DMO (two eyes) switched from faricimab back to aflibercept due to reported worse pain with faricimab injections. A further patient with DMO was switched to Ozurdex at six months due to disease progression (right eye VA gain of two ETDRS letters despite CST increase of 92 μm at six months; left eye VA gain of five letters with CST decrease of 29 μm at six months).

## Discussion

We present outcomes from a cohort of patients who had previously shown a partial response to anti-VEGF treatment and were switched to faricimab. This led to a significant improvement in anatomical outcomes, and enabled extension of treatment interval in approximately half of the eyes switched, with beneficial impact on the burden of visits and injections for both patients and healthcare services. Faricimab was well tolerated and no cases of endophthalmitis, retinal vasculitis or severe intraocular inflammation events were observed.

Outcome data from real-world studies on patients switched from other anti-VEGF agents to faricimab are now emerging and are summarised in Table 2 [23–47]. The majority show similar outcomes to our cohort, in that there were no significant safety concerns and VA remained largely stable following the switch. In addition to disease activity, VA is also affected by the level of degenerative change present at baseline and at follow up. Given that eyes in our cohort, and possibly others reported that were switched to faricimab, had chronic disease with a mean of over 20 injections prior to switching, the presence of photoreceptor degeneration may have limited potential VA gains. It is difficult to compare anatomical outcomes in all studies directly, since reports do not always state whether the indication for switching was the persistence of disease activity, or whether switching occurred primarily to extend treatment intervals, or to match the treatment given in the fellow eye. Furthermore, not all real-world studies have comprehensively reported adverse events to date, and most have a short follow-up period.

**Table 2:** Summary of real-world studies reporting outcomes from patients switched to faricimab [23–47].

Study	Eyes	Patients	Follow up (months)	Number of injections	VA Change* (ETDRS)	CST Change (μm)	Tx interval	Adverse events	Comments
**AMD**									
Rush 2023 [23]	54	54	12	>6	6.5	–45.4	31.5% reached an interval >8 weeks	Not reported	
Rush 2022 [24]	28	28	4	3	6.5	–64.6	Not reported	Not reported	
Khanani 2023 [25]	81	75	6	3	2.7	–38.1	Not reported	1 endophthalmitis, 1 anterior chamber inflammation	Outcomes after 3 injections
Grimaldi 2023 [26]	26	26	7.5	4	2.5	–65	Maximal fluid free interval increased from median 4 weeks to median 6 weeks	1 RPE tear	Outcomes after 4 injections weekly, an 8 week extension then treat and extend
Leung 2023 [27]	190	186	8	7	3	–25	Last average dosing interval 7.64 weeks	0	
Inoda 2023 [28]	80	75	NA	1	–1	–3.1^1^, +6.7^2^	NA	0	CST change for eyes switched from brolucizumab^1^ and aflibercept^2^
Kishi 2023 [29]	55	55	4	3	1.75**	–20**	Significantly extended from 5.9 to 7.5 weeks	1 RPE tear	** Approximate values derived from graph.
Kataoka 2023 [30]	130	124	6	4	0	–17.9	In 40.8% who continued, extended to 8.7 weeks	1 eye mild iritis.	59.2% switched away from faricimab due to worse exudation
								6 eyes macula haemorrhage	
Szigiato 2023 [31]	126	106	6	3	–0.2	–17	Interval extended from 5.7 to 6.3 weeks (not significant)	2 eyes (1 patient) intraocular inflammation	11 Eyes switched away from faricimab
Pandit 2023 [32]	218	191	NA	4	1.5	–48.2	Interval between third and fourth injection was 43.1 days compared to 35.7 days at baseline	2 RPE tears	Outcomes after 4 injections
Talks 2023 [33]	775	Not reported	NA	***	0.4	Not reported	Last mean treatment interval 7.4 weeks	Not reported	*** Between 5th and 6th injection shown
Raimondi 2023 [34]	81	68	NA	>6	0.1	-43.5	NA	0	
Goodchild 2024 [35]	98	79	>6 months	>6	–1	–45	75% of patients needing 4 weekly injections were extended	1 anterior uveitis, 1 endophthalmitis, 1 RPE rip	
Quah 2024 [36]	74	69	2.5	4.4	2.4	–57.3	NA	Not reported	
Muth 2024 [37]	57	48	1	NA	0.1	–27.9	NA	0	Outcome after 1 month
Aljundi 2024 [38]	33	33	12	8.6	2.5	–87	NA	0	
Eckardt 2024 [39]	58	51	Not reported	>3	–1.5	–151	Mean injection interval was comparable between baseline and	0	
Schneider 2024 [40]	50	46	1	NA	0	–31	NA	0	
Ng 2024 [41]	63	54	6,98	4.81	–1	–23.7	Clinical stable maintenance interval was extended to 5.25 weeks	2 patients had a conjunctival corneal ulcer and abrasion	Baseline interval was 5.24 with 69.8% previously on 4 weekly injections
								1 patient had an ischaemic stroke	
**DMO**									
Rush 2023 [42]	51	51	12	>6	6.5	–59.6	39.2% reached >8 weeks	None reported	
Rush 2022 [43]	24	24	4	3	5	–59.9	Not reported	None reported	
Bailey 2023 [44]	225	Not reported	NA	***	1.1	Not reported	Last mean treatment interval 7.2 weeks	Not reported	*** Between 5th and 6th injection shown
Pichi 2024 [45]	100	100	6	2.9	5	67.9	NA	Not reported	
Quah 2024 [36]	72	58	2.5	4.7	2.5	–104	NA	Not reported	
Tatsumi 2024 [46]	29	21	NA	4	-3.3	–53	NA	Not reported	
Durrani 2024 [47]	69	53	NA	3	–1	–57	25 eyes (36.2%) were able to be extended by 2 or more weeks	0	

*Data reported as logMAR or Snellen values have been converted to ETDRS letters to allow comparison.

*NA* not applicable.

Faricimab is the first bi-specific antibody approved for use in the eye, inhibiting both VEGF and Ang-2 pathways [48, 49], the latter via competitive inhibition of Ang1 binding to the Tie2 receptor, which would otherwise autophosphorylate, and this enhances vascular stability via P13K/AKT/ERK signalling [50, 51]. It is well established that multiple pathways are involved in the development nAMD and DMO [52, 53] and therefore in partial anti-VEGF responders it is feasible that blockade of an alternative pathway could mediate greater treatment effects. Previous studies examining outcomes following switch from ranibizumab (VEGF-A inhibitor) to aflibercept (VEGF-A and B and placental growth factor inhibitor) have shown improved effectiveness and responses [54, 55], although this effect was not long lasting in some reports [56]. Interestingly, switch from aflibercept to ranibizumab, the latter having a narrower spectrum of blockade, has also demonstrated beneficial effect in some patients [57]. Therefore, the impact of switching may be more complex than drug mechanism alone, but involve an interplay between this, dosing strategies and tachyphylaxis. Longer term outcomes in this group of patients switched to faricimab are awaited to determine the longevity of the responses seen.

A small number of eyes in our study showed worsening of disease following switch to faricimab. Two eyes with DMO were switched to Ozurdex at six months and one eye with nAMD was switched back to aflibercept at three months (Supplementary Fig. 1). In addition, a further two eyes with nAMD showed a more than 50 μm increase in CST at six months compared to baseline, and two eyes with DMO showed a >10% increase in CST at six months. These eight eyes represent <6% of the total switched to faricimab, indicating an overall beneficial effect of switching these difficult to treat eyes. However, these data highlight that mechanisms driving disease may differ between patients, and that a personalised approach may be required for treatment-resistant eyes.

There is currently debate among retina specialists as to whether patients switched to faricimab should be reloaded (i.e. receive a loading course of one injection of faricimab every 4 weeks for 4 injections), to maximise the effect of Ang-2 blockade, or whether it would be reasonable to maintain the current dosing interval at the time of switch, without a loading course of faricimab injections. In nAMD, where the intention was to match treatment intervals on switching, 25% of eyes were prescribed four injections at four-weekly intervals, whereas in the other 75% of eyes the treatment interval was extended more quickly, reducing injection burden. In DMO, the decision to reload or interval match on switching was dependent on the clinician, and 78% of eyes received four injections at four-weekly intervals. This should be taken into account when assessing the extension in treatment interval from 5.2 (±1.8) weeks at baseline to 6.6 (±2.5) weeks at six months (1.4 week difference, *p* < 0.05).

The strengths of this study are that we examined the outcomes of switching to faricimab in a real-world context, helping to address the evidence gap. In addition, this study provides information on a cohort of patients who have shown partial anti-VEGF response previously, with characterisation of anatomical measures of disease activity at the time of switching and six months’ follow-up data. Limitations are its retrospective nature and limited follow-up duration. Data collection is ongoing and, alongside other studies, will provide longer term evidence to assess the effectiveness and safety of switch to faricimab in nAMD and DMO.

## Conclusion

Switching to faricimab therapy in eyes that had responded poorly to other anti-VEGF agents led to stable visual outcomes, improved anatomical response, and extended treatment interval in a significant proportion of patients with nAMD and DMO at six months. Extending treatment intervals contributes to reducing the treatment burden for patients, and costs for healthcare systems. Longer term follow-up is needed to assess the durability of the treatment responses seen.

## Summary

### What was known before

- Landmark clinical trials demonstrated efficacy and safety of faricimab predominantly in treatment naïve patients.
- Data on patients switched to faricimab from other anti-VEGF agents are emerging, with the majority of studies reporting outcomes in patients with neovascular AMD (nAMD).

### What this study adds

- In our cohort comprising eyes treated for nAMD and those with diabetic macular oedema (DMO), an improved anatomical response was seen in a significant proportion of patients and visual acuity remained stable.
- Treatment interval was extended by a mean of 1.7 weeks in eyes with nAMD, and 1.4 weeks in eyes with DMO at six months. Extension of treatment interval was achieved in over half of the eyes switched, reducing treatment burden for patients and costs for healthcare systems.
- Longer term outcomes are needed to determine the durability of these effects.

## Supplementary information

Supplementary figure legends

Supplementary Figure 1

Supplementary Figure 2

Supplementary Figure 3

## Data Availability

The datasets generated and analysed in this study are not publicly available due to patient confidentiality, but anonymised data are available from the corresponding author on request.
