# Supplementary material for: Real-world six-month outcomes in patients switched to faricimab following partial response to anti-VEGF therapy for neovascular age-related macular degeneration and diabetic macular oedema
Source: Eye (Lond). 2024 Oct 11;38(18):3569–77. doi: 10.1038/s41433-024-03364-y (PMC11621343; doi:10.1038/s41433-024-03364-y)
Supplement: Supplementary file 1 — Supplementary figure legends [file 41433_2024_3364_MOESM1_ESM.docx]

**Supplementary Figure legends**

**Supplementary Figure 1.** Flowchart of inclusion and exclusion criteria.

**Supplementary Figure 2.** Treat and Extend Protocol used after switch to faricimab.(58)

**Supplementary Figure 3.** Correlation between glycated haemoglobin level (HbA1c, %) in patients with diabetic macular oedema and percentage change in central subfield thickness at six months following switch to faricimab.

**References:**

58. Bailey C, Cackett P, Kotagiri A, Mahmood S, Minos E, Narendran N, et al. Practical implementation of a q4-q16 aflibercept treat-and-extend pathway for the treatment of neovascular age-related macular degeneration: Updated guidance from a UK expert panel. Eye (Lond). 2023;37(9):1916-21.
