# Supplementary material for: Real-world six-month outcomes in patients switched to faricimab following partial response to anti-VEGF therapy for neovascular age-related macular degeneration and diabetic macular oedema
Source: Eye (Lond). 2024 Oct 11;38(18):3569–77. doi: 10.1038/s41433-024-03364-y (PMC11621343; doi:10.1038/s41433-024-03364-y)
Supplement: Supplementary file 2 — Supplementary Figure 1 [file 41433_2024_3364_MOESM2_ESM.pdf]

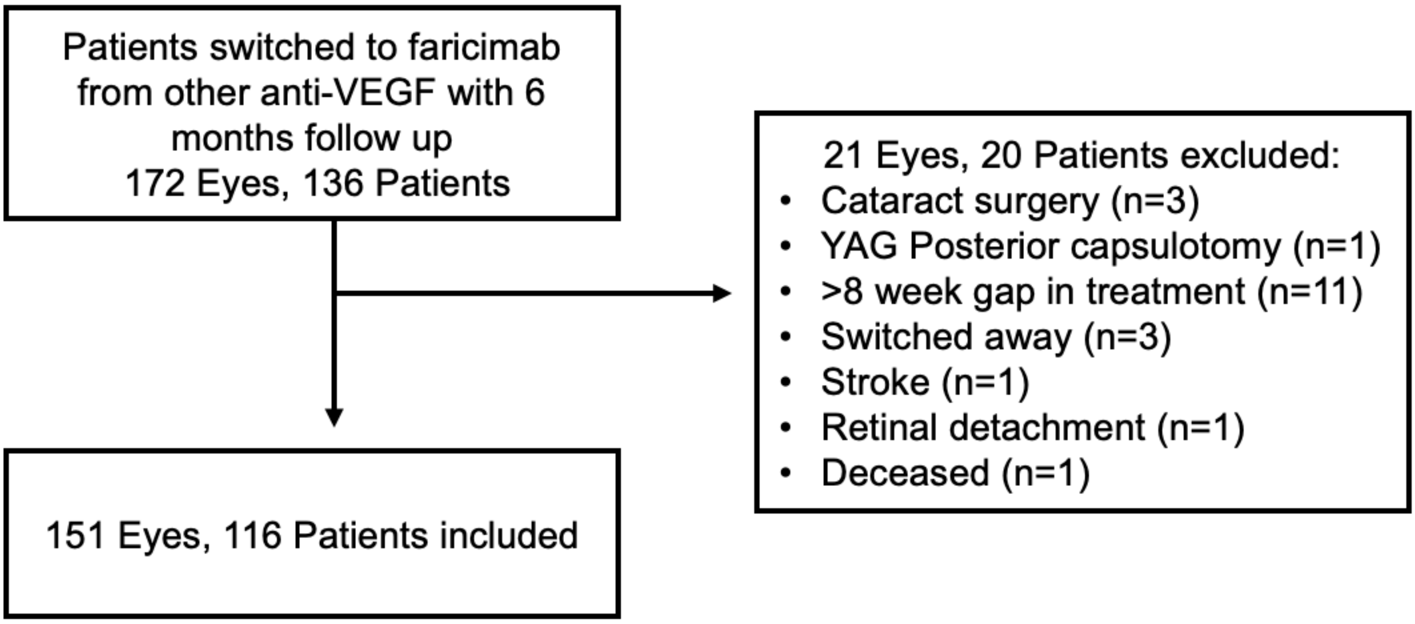
Patients switched to faricimab from other anti-VEGF with 6 months follow up
172 Eyes, 136 Patients
21 Eyes, 20 Patients excluded:
Cataract surgery (n=3)
YAG Posterior capsulotomy (n=1)
>8 week gap in treatment (n=11)
Switched away (n=3)
Stroke (n=1)
Retinal detachment (n=1)
Deceased (n=1)
151 Eyes, 116 Patients included
