# Supplementary material for: Real-world six-month outcomes in patients switched to faricimab following partial response to anti-VEGF therapy for neovascular age-related macular degeneration and diabetic macular oedema
Source: Eye (Lond). 2024 Oct 11;38(18):3569–77. doi: 10.1038/s41433-024-03364-y (PMC11621343; doi:10.1038/s41433-024-03364-y)
Supplement: Supplementary file 3 — Supplementary Figure 2 [file 41433_2024_3364_MOESM3_ESM.pdf]

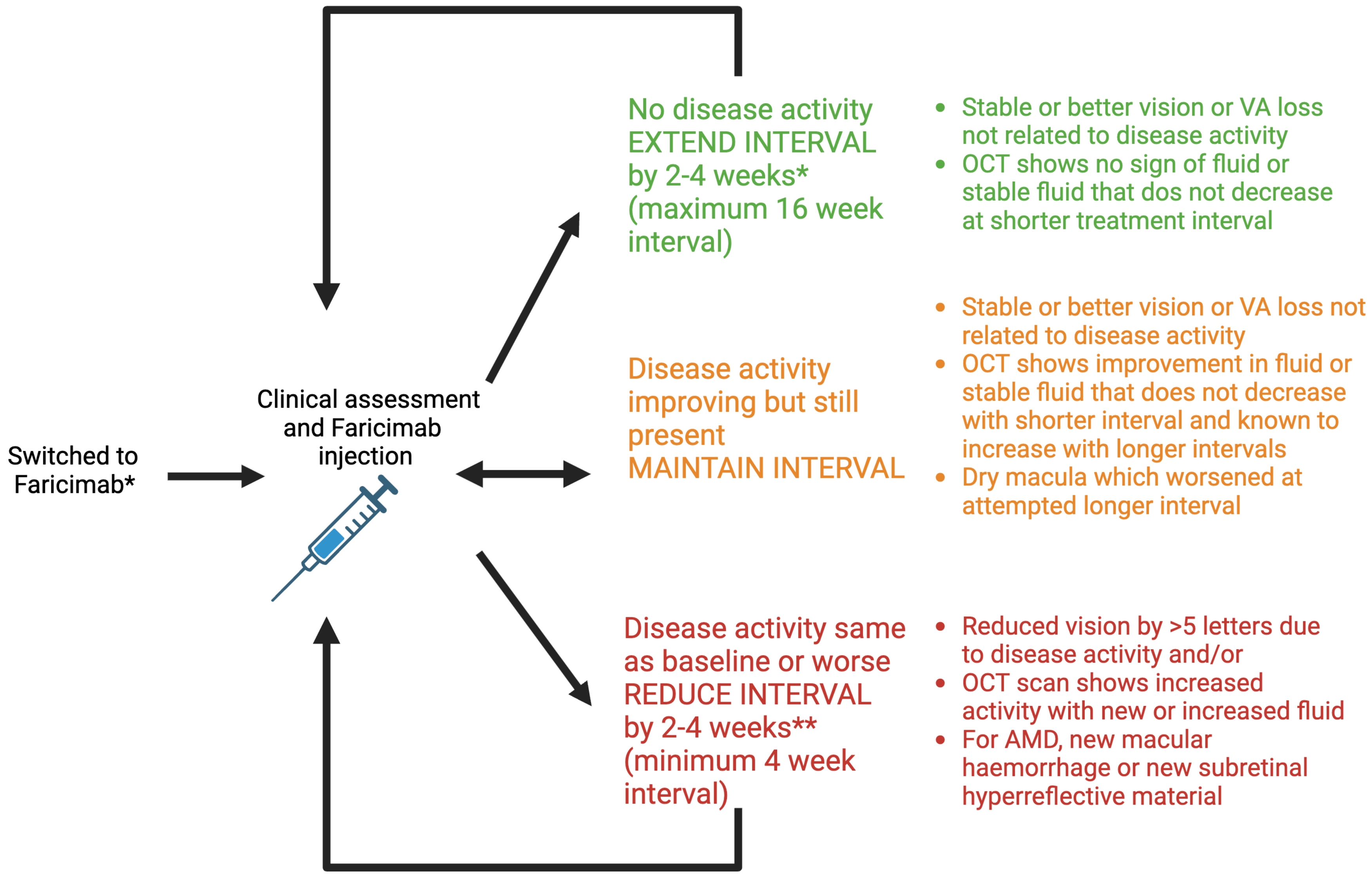

*see methods for switched protocol

**treatment intervals usually extended or reduced by 2 weeks in AMD and 4 weeks in DMO
